# Reduction in Cadmium Exposure in the United States Population, 1988–2008: The Contribution of Declining Smoking Rates

**DOI:** 10.1289/ehp.1104020

**Published:** 2011-11-07

**Authors:** Maria Tellez-Plaza, Ana Navas-Acien, Kathleen L. Caldwell, Andy Menke, Paul Muntner, Eliseo Guallar

**Affiliations:** 1 Department of Epidemiology, and; 2 Department of Environmental Health Sciences, Johns Hopkins Bloomberg School of Public Health, Baltimore, Maryland, USA; 3 Department of Epidemiology, Imaging and Atherothrombosis, National Center for Cardiovascular Research, Madrid, Spain; 4 Welch Center for Prevention, Epidemiology and Clinical Research, Johns Hopkins Medical Institutions, Baltimore, Maryland, USA; 5 Division of Laboratory Sciences, National Center for Environmental Health, Centers for Disease Control and Prevention, Atlanta, Georgia, USA; 6 Department of Epidemiology, University of Alabama at Birmingham, Birmingham, Alabama, USA; 7 Department of Medicine, Johns Hopkins Medical Institutions, Baltimore, Maryland, USA

**Keywords:** cadmium, cigarette smoking, determinants, NHANES, trends

## Abstract

Background: Public health policies such as tobacco control, air pollution reduction, and hazardous waste remediation may have reduced cadmium exposure among U.S. adults. However, trends in urine cadmium, a marker of cumulative cadmium exposure, have not been evaluated.

Objectives: We estimated the trends in urine cadmium concentrations in U.S. adults using data from the National Health and Nutrition Examination Surveys (NHANES) from 1988 to 2008. We also evaluated the impact of changes in the distribution of available cadmium determinants (age, sex, race, education, body mass index, smoking, and occupation) at the population level to explain cadmium trends.

Methods: The study population included 19,759 adults ≥ 20 years of age with measures of urine cadmium and cadmium determinants.

Results: Age-adjusted geometric means of urine cadmium concentrations were 0.36, 0.35, 0.27, 0.27, 0.28, 0.25, and 0.26 µg/g creatinine in 1988–1991, 1991–1994, 1999–2000, 2001–2002, 2003–2004, 2005–2006, and 2007–2008, respectively. The age, sex, and race/ethnicity-adjusted percent reduction in urine cadmium geometric means comparing 1999–2002 and 2003–2008 with 1988–1994 were 27.8% (95% confidence interval: 22.3%, 32.9%) and 34.3% (29.9%, 38.4%), respectively (*p*-trend < 0.001), with reductions in all participant subgroups investigated. In never smokers, reductions in serum cotinine accounted for 15.6% of the observed reduction. In ever smokers, changes in smoking cessation, and cumulative and recent dose accounted for 17.1% of the observed reduction.

Conclusions: Urine cadmium concentrations decreased markedly between 1988 and 2008. Declining smoking rates and changes in exposure to tobacco smoke may have played an important role in the decline of urine cadmium concentrations, benefiting both smokers and nonsmokers. Cadmium has been associated to several health outcomes in NHANES 1999–2008. Consequently, despite the observed decline, further reduction in cadmium exposure is needed.

Cadmium is a highly toxic and carcinogenic metal widely distributed in the environment. Mounting evidence from general populations exposed to low or moderate levels points to cadmium as a risk factor for a broad spectrum of health conditions, including cardiovascular, kidney, and bone disease (Järup and Akesson 2009; Nordberg et al. 2007; Satarug et al. 2010). Smoking, diet (leafy and root vegetables, grains, and offal), ambient air, and occupation exposures (metal and mining industry, transportation, and repairing services) are the main sources of cadmium exposure (Järup and Akesson 2009; Nordberg et al. 2007; Satarug et al. 2010; Yassin and Martonik 2004). Public health policies such as tobacco control [Breysse and Navas-Acien 2010; U.S. National Center for Health Statistics (NCHS) 2010b], air pollution reduction [U.S. Environmental Protection Agency (EPA) 2010a, 2010b], and hazardous waste remediation (U.S. EPA 2010c) may have resulted in decreased cadmium exposure in the U.S. population. In the U.S. population, however, trends in cadmium exposure over time have not been evaluated.

The National Health and Nutrition Examination Survey (NHANES) provides periodic monitoring of the health of the U.S. population. Since 1988, NHANES has included the measurement of cadmium concentrations in urine (NCHS 2010a). Urine cadmium is a biomarker of cumulative cadmium exposure and internal dose that reflects cadmium concentrations in the renal cortex (Järup and Akesson 2009; Nordberg et al. 2007). Our objective was to estimate trends in cadmium exposure, as measured by urine cadmium, in the general U.S. population from 1988 to 2008. In addition, we evaluated the impact of population changes in the distribution of cadmium determinants, in particular smoking, to explain changes in urine cadmium concentrations over time.

## Materials and Methods

*Study population.* NHANES uses a complex multistage sampling design to obtain representative samples of the noninstitutionalized U.S. population (NCHS 2010a). We used data from NHANES III (1988–1994), collected in two phases (1988–1991 and 1991–1994), and from NHANES 1999–2008, collected in five phases (1999–2000, 2001–2002, 2003–2004, 2005–2006, and 2007–2008). In NHANES III, urine cadmium was measured in all participants ≥ 6 years of age. In NHANES 1999–2008, urine cadmium was measured in a random one-third subsample of participants ≥ 6 years of age. For this analysis we included 23,904 adults ≥ 20 years of age (7,967 in 1988–1991, 8,169 in 1991–1994, 1,299 in 1999–2000, 1,560 in 2001–2002, 1,532 in 2003–2004, 1,520 in 2005–2006, and 1,857 in 2007–2008). We excluded 628 pregnant women; 109 participants with missing urine creatinine measurements; 3,179 participants with missing smoking status, pack-years, or serum cotinine; and 229 participants with missing other variables of interest. A total of 19,759 participants (6,616 in 1988–1991, 7,075 in 1991–1994, 965 in 1999–2000, 1,166 in 2001–2002, 1,209 in 2003–2004, 1,160 in 2005–2006, and 1,568 in 2007–2008) were included in our analyses. Participants included in this analysis were similar to the corresponding NHANES-phase population with respect to sociodemographic variables (data not shown).

*Urine cadmium.* Cadmium in spot urine samples was measured at the Environmental Health Laboratory of the Centers for Disease Control and Prevention’s (CDC) National Center for Environmental Health (Atlanta, GA, USA) for all surveys. Extensive quality control procedures were followed, including confirmation that collection and storage materials were not contaminated with background cadmium or other metals (NCHS 2010a).

Urine cadmium was measured by graphite furnace atomic absorption spectrometry (model 3030; PerkinElmer, Norwalk, CT, USA) with Zeeman background correction in NHANES 1988–1994, by inductively coupled plasma mass spectrometer (ICP-MS; ELAN, PerkinElmer) in NHANES 1999–2002, and by ICP–dynamic reaction cell (DRC)-MS (ELAN DRC, PerkinElmer) in NHANES 2003–2008 (NCHS 2010a). In NHANES 1988–1994, specimens were analyzed in duplicate, and the average of the two measurements was reported (Paschal et al. 2000). The interassay coefficients of variation ranged from 2.8% to 13.6%, and the limit of detection (LOD) was 0.03 µg/L (NCHS 2010a), resulting in 6% of observations below the LOD. In NHANES 1999–2008, the interassay coefficients of variation for urine cadmium ranged from 1.2% to 6.7%, and the LOD was 0.06 µg/L in 1999–2004 and 0.042 µg/L in 2005–2008, resulting in 4% of observations below the LOD. In all phases, the urine reference material from the National Institute of Standards and Technology (NIST 2010) was analyzed periodically to ensure analytical accuracy, and observed concentrations were in good agreement with published values (96–101%) (Jarrett et al. 2008; Paschal et al. 2000). Two levels of in-house urine pools traceable to the reference material were used for daily quality control. One of two different levels of a blind quality control material was inserted in every analytical group of samples for an additional quality control check. In NHANES III, laboratory measures were within 10% of reference means for urinary cadmium (*r*^2^ = 0.97) (Paschal et al. 2000). Precision data for NHANES 1999–2008 are discussed in detail in Jarrett et al. (2008). In brief, NHANES 1999–2002 data were mathematically adjusted to eliminate a bias in the original standard-mode quadrupole ICP-MS data caused by a molybdenum-based interference. The CDC lab used a new ICP-DRC-MS method starting in the NHANES 2003–2004 cycle to eliminate the molybdenum oxide interference. Therefore, starting with the 2003–2004 survey cycle, the need for mathematical correction of the urine cadmium data was eliminated (Jarrett et al. 2008). For observations below the LOD and for values corrected for interference from molybdenum oxide equal to 0 in 1999–2002 data (*n* = 4), urine cadmium value was imputed as the LOD divided by the square root of 2 (Hornung and Reed 1990).

Creatinine-corrected urine cadmium data were reported in micrograms cadmium per gram creatinine. Urine creatinine was measured by the modified kinetic Jaffé method in 1988–2006 and by an enzymatic (creatinase) method in 2007–2008 (NCHS 2010a). We corrected urine creatinine determinations before 2007 as recommended by NHANES (NCHS 2010a).

*Urine cadmium determinants.* Information on age, sex, race/ethnicity, education, smoking status, cigarette pack-years, occupation, body mass index (BMI), and serum cotinine was based on questionnaires, physical examination, and laboratory methods that have been described elsewhere (Benowitz et al. 1983; NCHS 2010a; Yassin and Martonik 2004). We classified study participants as current smokers if they answered yes to the question “Do you smoke cigarettes now?” or had serum cotinine levels > 10 ng/mL (Benowitz et al. 1983). Former smokers were participants who answered yes to the question “Have you smoked at least 100 cigarettes during your entire life?” but were not current smokers. Pack-years were determined using answers to the following questions: “How old were you when you first started smoking cigarettes fairly regularly?”, “About how many cigarettes do you smoke per day now?” (or, because of changes in the smoking questionnaires in NHANES 2007–2008, “Average number of cigarettes/day during the past 30 days”), “For approximately how many years have you smoked this amount?”, “About how old were you when you last smoked cigarettes (fairly regularly)?”, and “About how many cigarettes per day did you usually smoke at that time?”

Occupations and industries associated with cadmium exposure were based on the study by Yassin and Martonik (2004) and included self-reported occupations related to transportation, metal, mining, and repairing service industries. Duration of the longest cadmium-associated occupation was determined using answers to the questions: “What kind of work were you doing (in the past 1 or 2 weeks, depending on the survey period)?”, “About how long have you worked for (employer) as a(n) (occupation) (in the past 1 or 2 weeks, depending on the survey period)?”, “Thinking of all the paid jobs or businesses you ever had, what kind of work were you doing the longest?”, and “About how long did you work at that job or business (the longest)?” For participants with the longest held job not being cadmium related, the current job was also considered as a potential source of cadmium. Information on occupation was not available in NHANES 2005–2008.

Serum cotinine was measured by an isotope-dilution high-performance liquid chromatography/atmospheric pressure chemical ionization tandem MS method (NCHS 2010a). The LOD for serum cotinine was 0.05 ng/mL in NHANES III and 0.015 ng/mL in NHANES 1999–2008. For the 4.9% and 9.0% of participants below the LOD in NHANES III and NHANES 1999–2008, respectively, cotinine concentrations were replaced by the LOD divided by the square root of 2.

*Statistical methods.* Statistical analyses were performed using the survey package (Lumley 2004) in R software (version 2.12.1; R Development Core Team 2011) to account for the complex sampling design and weights in NHANES 1988–2008 and to obtain appropriate standard errors for all estimates. Urine cadmium levels were right skewed and were log transformed for the analyses. Age-adjusted cadmium concentrations were obtained using the residuals from linear regression models of log-urine cadmium concentrations corrected for creatinine modeled by age as restricted cubic splines with 5 knots, and adding back the cadmium-weighted means of the corresponding NHANES samples. Age-adjusted creatinine-corrected urine cadmium concentrations were reported as geometric means.

Starting in 1999, urine cadmium was measured in a one-third random subsample of NHANES participants only. To increase the sample size in regression models over time, we grouped multiple phases together (1988–1994, 1999–2002, and 2003–2008). Temporal trends in urine cadmium concentrations were evaluated by estimating geometric mean ratios and 95% confidence intervals (CIs) of urine cadmium concentrations in NHANES 1999–2002 and NHANES 2003–2008 compared with NHANES 1988–1994. The geometric mean ratios were obtained by exponentiating linear combinations of β-coefficients in regression models adjusted for age, sex, and race/ethnicity with log-transformed cadmium as the dependent variable and survey phase group and cadmium determinants as interacting independent variables. Subsequently, percent reductions in geometric means over time [estimated as (1 – geometric mean ratio) × 100] and 95% CIs were calculated overall and by subgroups of cadmium determinants. We considered the following subgroups of cadmium determinants: age (< 35, 35–49, 50–65, and ≥ 65 years), sex (men and women), race/ethnicity (white, African American, Mexican American, and other), education (< high school and high school, or higher education), BMI (< 25, 25–30, and ≥ 30 kg/m^2^), cigarette smoking status (never, former, current), cigarette pack-years (0, 0–10, 10–20, and > 20 pack-years), serum cotinine (< 0.05, 0.05–10, 10–200, ≥ 200 ng/mL), and duration of the longest occupation associated to cadmium (0, 0–10, 10–20, > 20 years in the subset of participants with information available). We estimated *p*-trend values by applying the Wald test to the appropriate regression coefficients.

Toxicokinetic parameters related to cadmium absorption, accumulation in the renal cortex, and excretion, such as low iron stores or reduced kidney function, could induce variation in urine cadmium concentrations independently of variations in exposure (Amzal et al. 2009; Ruiz et al. 2010). Therefore, we performed a sensitivity analysis using models adjusted for serum iron, and glomerular filtration rate, in addition to age, sex, and race/ethnicity. Results were similar to those reported here (data not shown).

The relative contribution of cadmium determinants to the trend in urine cadmium concentrations was calculated as the relative change in the β-coefficient for survey phase category (grouped as 1999–2008 vs. 1988–1994) in regression models for log-transformed cadmium after sequentially introducing cadmium determinants in linear regression models. In this analysis, NHANES 1999–2002 and 2003–2008 were combined and compared with NHANES 1988–1994 for simplicity, because results in NHANES 1999–2002 and NHANES 2003–2008 separately yielded similar findings. Because occupation variables were available only until 2004, the contribution of occupation to the change in urine cadmium concentrations was evaluated only for the available period.

We also conducted a sensitivity analysis comparing estimates from models of urine cadmium concentrations (micrograms per liter) adjusted for log-transformed urine creatinine (data not shown) with those from models of creatinine-corrected urine cadmium concentrations (micrograms per gram). Results were comparable to those reported here.

## Results

*Participant characteristics.* Between 1988 and 2008, the U.S. population became older and more educated, and its average BMI increased [see Supplemental Material, Table 1 (http://dx.doi.org/10.1289/ehp.1104020)]. The prevalence of never smokers increased from 46.0% in 1988–1994 to 53.8% in 2003–2008, whereas cigarette pack-years (mean, 12.9 vs. 10.1 pack-years) and serum cotinine concentrations (geometric mean, 1.44 vs. 0.34 ng/mL) decreased markedly in the overall population.

**Table 1:** Urine cadmium levels over time by participant characteristics.*^a,b^*

Urine cadmium, geometric mean (µg/g)					Percent reduction of geometric mean (CI)*c*		
					1999–2002 vs. 1988–1994	2003–2008 vs. 1988–1994
Characteristic	*n*	1988–1994	1999–2002	2003–2008		
Overall		19,759		0.37		0.27		0.24		27.8 (22.3, 32.9)		34.3 (29.9, 38.4)
Age group (years)												
< 35		5,676		0.2		0.15		0.14		23.0 (14.9, 30.3)		30.5 (23.7, 36.8)
35–50		5,231		0.36		0.27		0.25		25.0 (16.0, 33.0)		32.2 (25.2, 38.6)
50–65		4,045		0.6		0.41		0.37		31.9 (24.1, 38.9)		38.8 (33.3, 43.8)
≥ 65		4,807		0.69		0.44		0.44		35.5 (30.3, 40.2)		35.6 (30.4, 40.4)
Sex												
Men		9,360		0.31		0.22		0.21		29.0 (22.8, 34.7)		33.5 (28.5, 38.1)
Women		10,399		0.44		0.32		0.28		26.7 (20.3, 32.6)		35.0 (29.9, 39.7)
Race/ethnicity												
White		8,734		0.36		0.26		0.24		27.5 (20.3, 34.0)		33.2 (27.4, 38.5)
African American		4,830		0.41		0.27		0.24		35.1 (26.1, 43.1)		42.2 (38.2, 46.0)
Mexican American		5,061		0.38		0.29		0.26		23.2 (14.7, 30.9)		32.8 (27.2, 37.9)
Other		1,134		0.44		0.33		0.29		25.3 (13.0, 35.8)		34.3 (23.6, 43.5)
Education												
≥ High school		12,485		0.35		0.26		0.24		26.7 (20.6, 32.3)		32.1 (27.2, 36.6)
< High school		7,274		0.46		0.33		0.29		28.7 (21.6, 35.2)		37.1 (31.7, 42.0)
BMI (kg/m^2^)												
< 25		7,217		0.38		0.31		0.27		19.3 (8.8, 28.5)		28.8 (21.9, 35.2)
25–30		6,996		0.38		0.26		0.24		31.4 (24.7, 37.4)		35.0 (28.8, 40.7)
≥ 30		5,546		0.35		0.24		0.22		30.9 (24.4, 36.9)		37.4 (32.7, 41.8)
Smoking												
Never		10,107		0.27		0.21		0.19		22.1 (15.4, 28.4)		28.4 (22.7, 33.8)
Former		4,524		0.38		0.27		0.25		29.8 (22.3, 36.5)		35.7 (29.8, 41.0)
Current		5,128		0.6		0.47		0.41		22.2 (13.7, 29.9)		32.4 (26.4, 37.8)
Pack-years												
0		10,177		0.26		0.21		0.19		21.1 (14.5, 27.1)		27.0 (21.3, 32.3)
0–10		4,075		0.34		0.28		0.23		16.6 (5.8, 26.2)		31.2 (24.5, 37.3)
10–20		1,803		0.51		0.37		0.34		26.3 (14.8, 36.3)		32.5 (24.3, 39.9)
> 20		3,704		0.71		0.5		0.49		29.8 (23.1, 36.0)		31.4 (25.7, 36.8)
Serum cotinine (ng/mL)												
< 0.05		4,578		0.25		0.22		0.2		13.3 (3.0, 22.5)		20.3 (11.1, 28.6)
0.05–10		10,328		0.32		0.24		0.22		25.3 (17.9, 32.1)		31.0 (25.5, 36.2)
10–200		2,062		0.45		0.4		0.34		11.3 (–1.8, 22.7)		24.2 (14.1, 33.1)
≥ 200		2,791		0.76		0.57		0.47		24.1 (15.0, 32.3)		38.2 (32.5, 43.3)
Duration of longest cadmium-associated occupation (years)*d*												
0		17,480		0.36		0.26		0.25		26.9 (21.0, 32.3)		30.1 (24.1, 35.6)
0–10		3,904		0.43		0.32		0.33		27.1 (9.3, 41.3)		23.9 (11.1, 34.9)
10–20		3,476		0.47		0.35		0.35		26.2 (4.3, 43.1)		26.2 (0.5, 45.2)
> 20		3,578		0.51		0.34		0.31		33.5 (15.4, 47.8)		39.7 (23.7, 52.4)
**a**Geometric means and percent reductions in geometric means were adjusted for age (years modeled as restricted cubic splines with 5 knots), sex (men, women), and race/ethnicity (white, African American, Mexican American, and other). Geometric means were further recalibrated to overall mean. **b***p*-Trend was < 0.001 overall and for all subgroups. **c**We obtained adjusted ratios of geometric means comparing survey phase groups (NHANES 1999–2002 and 2003–2008) with respect to the reference survey phase group (NHANES 1988–1994) by exponentiating linear combinations of β-coefficients from regression models with log-transformed cadmium as the dependent variable and survey phase group and confounder factors as interacting independent variables. Subsequently, the percent reduction of the geometric mean was estimated as (1 – ratio of geometric mean) × 100. For instance, a 28.7% reduction comparing urine cadmium concentrations in NHANES 1999–2002 to NHANES 1988–1994 corresponded to a ratio of geometric means comparing NHANES 1999–2002 with NHANES 1988–1994 equal to 0.713. *p*-Trends comparing 1999–2002 and 2003–2008 with 1988–1994 were obtained using the Wald test. **d**Occupation variables were only available through 2004.												

*Cadmium determinants.* Age-, sex-, and race/ethnicity-adjusted geometric mean urine cadmium concentrations were higher with increasing age, in women compared with men, in former and current smokers compared with never smokers, with increasing cigarette pack-years and serum cotinine concentrations, and in participants with a cadmium-associated occupation during all three time periods [1988–1994, 1999–2002, 2003–2008; see Supplemental Material, Table 2 (http://dx.doi.org/10.1289/ehp.1104020)]. The strength of the association between determinants and urine cadmium concentrations was similar across survey periods for all variables except race/ethnicity (African-American participants had increased urine cadmium concentrations compared with whites in 1988–1994 but not in 2003–2008).

**Table 2:** Percent reduction (95% CI) in urine cadmium geometric means comparing 1999–2008 and 1988–1994 in models with progressive degrees of adjustment.

Model adjustments	Overall (*n* = 19,759)	Never smokers (*n* = 10,107)	Ever smokers (*n* = 9,652)
Age		31.3 (26.9, 35.5)		24.9 (18.9, 30.5)		31.4 (27.1, 35.5)
Age, sex, race, education, and BMI		29.5 (25.1, 33.6)		23.7 (18.2, 28.8)		29.8 (25.3, 34.0)
Age, sex, race, education, BMI, and smoking status		27.2 (22.9, 31.2)		NA		30.5 (26.2, 34.5)*b*
Age, sex, race, education, BMI, smoking status, pack-year, and cotinine		21.7 (17.4, 25.9)		20.0 (14.1, 25.6)*c*		24.7 (20.4, 28.8)*b*
NA, not applicable. **a**The change in the percent reduction in geometric means comparing urine cadmium concentrations in NHANES 1999–2008 with those in NHANES 1988–1994 after introducing a given variable (or set of variables) is interpreted as the amount of the reduction in urinary cadmium that can be attributed to that variable (or set of variables). For instance, for the overall population, the age-adjusted percent reduction of geometric mean of urine cadmium in NHANES 1999–2008 compared with 1988–1994 was 31.3%. After further adjustment for age, sex, race, education, and BMI, the percent reduction was 29.5%; that is, changes in those variables explained 5.7% of the age-adjusted percent reduction [1 – (29.5/31.3) × 100 = 5.7%]. **b**Smoking status in ever smokers only included former and current smokers. **c**Adjusted for serum cotinine only in analysis restricted to never smokers.						

**Figure 1:**
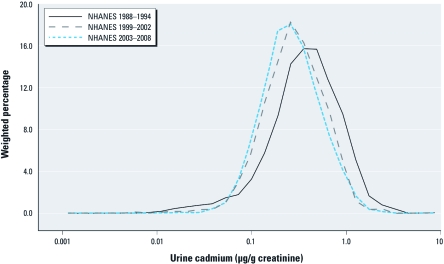
Distribution of age-adjusted urine cadmium in NHANES 1988–1994, 1999–2002, and 2003–2008. Urine cadmium concentrations were adjusted for age (years modeled as restricted cubic spline with 5 knots) in survey-period–specific strata.

*Cadmium trends.* The distribution of age-adjusted urine cadmium concentrations shifted progressively downward over time (Figure 1). Age-adjusted geometric mean urine cadmium concentrations were 0.36, 0.35, 0.27, 0.27, 0.28, 0.25, and 0.26 µg/g creatinine in 1988–1991, 1991–1994, 1999–2000, 2001–2002, 2003–2004, 2005–2006, and 2007–2008, respectively. The decrease in age-adjusted geometric mean of urine cadmium concentrations was observed in both men and women and among never, former, and current smokers [see Supplemental Material, Figure 1, Table 3 (http://dx.doi.org/10.1289/ehp.1104020)]. The overall prevalence of urine cadmium concentrations > 1 µg/g g [2009 tolerable weekly intake reference point, European Food Safety Authority Panel (EFSA) 2011)] was 16.04%, 4.53%, and 4.56% in 1988–1994, 1999–2002, and 2003–2008, respectively, although the observed decline was progressive over the study period in most subgroups, for example, in men, whites, African Americans, Mexican Americans, and never and current smokers (see Supplemental Material, Table 4).

Compared with 1988–1994, the age-, sex-, and race/ethnicity-adjusted percent reductions in the geometric means of urine cadmium concentrations were 27.8% and 34.3% in 1999–2002 and 2003–2008, respectively (Table 1), with reductions observed in all participant subgroups investigated. Older participants, African Americans, participants with less than high school education, obese participants, and former smokers and smokers in the highest cigarette pack-year and serum cotinine categories showed larger percent reductions compared with other groups.

*Contribution of cadmium determinants to the trend.* The reduction in the geometric mean of urine cadmium concentration between 1999–2008 and 1988–1994 was attenuated after additional adjustment for smoking status, pack-years, and serum cotinine [21.7% (95% CI: 17.4%, 25.9%) compared with 29.5% (25.1%, 33.6%) when adjusted for age, sex, race/ethnicity, education, and BMI only; Table 2]. Smoking-related variables thus accounted for 26.4% of the observed reduction in urine cadmium concentrations comparing the fully adjusted model with and without smoking variables ([1 – (21.7/29.5)] × 100). In analyses restricted to never smokers, serum cotinine concentrations accounted for 15.6% of the corresponding reduction in urine cadmium concentrations (23.7% vs. 20.0% reduction before and after adjustment for serum cotinine). In ever smokers (i.e., current and former smokers), smoking-related variables accounted for 17.1% of the corresponding reduction in urine cadmium concentrations (29.8% vs. 24.7% reduction before and after adjustment for current and former smoking status, pack-years, and serum cotinine). The coefficient of determination (*R*^2^) for the fully adjusted model was 0.46. Changes in cadmium-related occupations did not explain cadmium exposure trends in the subsample of the population with occupational data available, as results did not change before and after adjustment for occupation variables (data not shown).

## Discussion

Urine cadmium concentrations decreased by 34.3% from 1988–1994 to 2003–2008 in the U.S. adult population after adjustment for age, sex, and race/ethnicity. The reduction in adjusted urine cadmium concentrations was observed in all population subgroups evaluated, but it was stronger in ever smokers, heavier smokers, and African Americans compared with other subgroups. We identified the reduction in smoking rates as a key factor driving the reduction in urine cadmium over time. A large proportion of the reduction in urine cadmium concentrations, however, remained unexplained and could be related to changes in cadmium concentrations in ambient air, food, or other determinants not available in our survey. Given the multiple health consequences of cadmium exposure (Järup and Akesson 2009; Nordberg et al. 2007; Satarug et al. 2010), the decline of urine cadmium levels documented in the present analysis is an important public health achievement. Cadmium exposure, however, is still too high, because cadmium in the environment is mostly coming from anthropogenic sources [Agency for Toxic Substances and Disease Registry (ATSDR) 1999], and cadmium production was practically nonexistent as recently as the beginning of the 20th century [U.S. Geological Survey (USGS) 2010].

Cadmium was discovered in the 19th century (USGS 2010). A by-product from mining, from smelting, and from refining zinc, lead, and copper ores, cadmium industrial production started in the 1930s [ATSDR 1999; International Agency for Research on Cancer (IARC) 1993; USGS 2010]. Since then, the use of cadmium in consumer products (e.g., pigments, batteries, coatings, and plastic stabilizers) increased dramatically until the 1970s and 1980s (ATSDR 1999; Nordberg et al. 2007; USGS 2010), resulting in widespread soil contamination from industrial releases, fuel combustion, and cadmium-containing phosphate fertilizers (ATSDR 1999; Lalor 2008; Nordberg et al. 2007; Staessen et al. 1992). Soil contamination by cadmium is a major environmental health problem because leafy and root vegetables and grains bioconcentrate cadmium from soil (Dal Corso et al. 2008; Huang et al. 2008), providing a major pathway for exposure through diet and tobacco (Golia et al. 2009; Lalor 2008; Nordberg et al. 2007). Ambient air and dust can also contribute to cadmium exposure, particularly in urban areas and in the vicinity of industrial sources and waste sites (Hogervorst et al. 2007; Nordberg et al. 2007).

Urine cadmium concentrations in U.S. adults were lower than concentrations measured in northeastern Belgium (Nawrot et al. 2008) and nationwide surveys from Canada (Wong and Lye 2008), Japan (Ikeda et al. 2000), and Korea (Lee et al. 2010) but similar to concentrations in a nationwide survey from Germany (Schulz et al. 2007). Data on cadmium trends in population-based samples are scarce (Nawrot et al. 2008; Schulz et al. 2007). In northeastern Belgium, 24-hr urine cadmium concentrations decreased by 12.9% from 1985 to 1996, and blood cadmium concentrations decreased by 20% from 1985 to 2001–2003 (Nawrot et al. 2008). In Germany, the geometric mean urine cadmium concentrations decreased from 0.29 µg/L in 1990 to 0.24 µg/L in 1998, but no change was observed for blood cadmium (Schulz et al. 2007). In addition to urine, blood cadmium has been measured in NHANES participants since 1999. Given the lack of blood cadmium determinations in NHANES 1988–1994, the trend in blood cadmium concentrations from 1988 to 2008 could not be evaluated in our study population.

Tobacco-control policies in recent decades have resulted in important reductions in smoking prevalence and tobacco dose in the United States (NCHS 2010b; Pierce et al. 2011). Our study suggests that changes in smoking status (never, former, current), cumulative dose (cigarette pack-years), and recent dose (serum cotinine) have played an important role in the decline of urine cadmium concentrations in the U.S. population, benefiting both smokers and never smokers. Among ever smokers, changes in smoking variables accounted for 17.1% of the observed reduction in urine cadmium concentrations comparing fully adjusted models with and without smoking variables. Cumulative and recent smoking dose mostly contributed to the observed reduction. Furthermore, heavier smokers (higher categories of pack-years and serum cotinine concentrations) presented larger urine cadmium reductions. These findings could be due to the decrease in the number of cigarettes smoked per day (O’Connor et al. 2006; Pierce et al. 2011) and to changes in cigarette composition over time (Hoffmann et al. 2001; Scherer and Barkemeyer 1983). Among never smokers, changes in serum cotinine, a marker of exposure to secondhand smoke, accounted for 15.6% of the adjusted reduction in urine cadmium concentrations comparing fully adjusted models with and without cotinine. Cadmium is present both in sidestream and mainstream tobacco smoke (Chang et al. 2005; Kalcher et al. 1993; Pappas et al. 2006). Although data on secondhand smoke exposure as a source of cadmium exposure are scarce and inconsistent (Bolte et al. 2008; Conrad et al. 2010; McElroy et al. 2007), our results suggest that reductions in secondhand smoke exposure in recent decades have decreased cadmium exposure in U.S. adults. Additional tobacco-control efforts may further reduce cadmium exposure in the population, including legislating and promoting smoke-free environments, increasing cessation measures to help current smokers to quit, and preventing smoking initiation among adolescents and young adults.

Associations of urine cadmium with determinants other than smoking were also consistent with those in other populations, including higher cadmium concentrations with increasing age and among women, individuals with less than a high school education, and individuals with occupations related to cadmium exposure (Nordberg et al. 2007; Vahter et al. 2007). BMI showed an inverse association with urine cadmium concentrations. This association has been previously reported (Dhooge et al. 2010; Friedman et al. 2006; Padilla et al. 2010), although there is limited evidence providing a biological explanation for this finding. Changes in these determinants over time, however, contributed little to the reduction in urine cadmium concentrations. Occupation, for instance, is a modifiable source of cadmium exposure that was associated with urine cadmium concentrations in all study surveys but did not explain changes in urine cadmium concentrations over time. Given the well-described health effects of cadmium in occupational settings (ATSDR 1999; IARC 1993; Nordberg et al. 2007), additional exposure prevention efforts, including the implementation of the Occupational Safety and Health Administration recommendations for cadmium-associated occupations, are important to reduce and prevent cadmium exposure in occupational settings (U.S. Department of Labor 2010).

In subgroup analysis, African-American participants and participants with less than high school education presented larger reductions compared with other subgroups. For African Americans, urine cadmium concentrations changed from being higher than concentrations in whites in 1988–1994 to being similar to concentrations in whites in 2003–2008. For individuals with less education, urine cadmium concentrations in 2003–2008 remained higher than concentrations in more educated individuals. We could not evaluate the contribution of changes in cadmium exposure through diet and ambient air, because long-term individual exposure to cadmium from diet cannot be estimated from NHANES 24-hr recall questionnaires, and data on cadmium exposure information from air are not available. The U.S. EPA air pollution program did not systematically monitor air cadmium levels in cities over the entire study period, but there is evidence that cadmium production and particulate matter emissions from metal-processing industries started to decrease in the United States in the 1970s (U.S. EPA 2010b; USGS 2010). Nationwide environmental monitoring programs have shown a progressive decline in cadmium concentrations in biological specimens, in sediments, and in great lakes and coastal waters (O’Connor and Lauenstein 2006; U.S. Center for Coastal Monitoring and Assessment 2010; U.S. EPA 2011). Cadmium content in U.S. food markets has also been decreasing since 1990 (U.S. Food and Drug Administration 2010). It is thus possible that part of the reduction in urine cadmium concentrations found in this study was related to an overall decrease in environmental and dietary cadmium concentrations. Future studies monitoring cadmium exposure and its determinants should incorporate individual data on exposure from diet and ambient air. It is also important to evaluate the environmental impact and contribution to human exposure of nonrecycled cadmium-containing products (e.g., batteries, electronic devices, jewelry, and toys) and of cadmium-containing fertilizers (Mead 2010; USGS 2010).

In addition to the lack of information on cadmium exposure through diet and air, it is possible that changes in laboratory methods over time could have affected the observed trends in urine cadmium levels. Specially, changes in laboratory methods could be partly responsible of the residual trend that could not be attributed to the available determinants. However, urine cadmium was measured in the same laboratory under strict quality control measures with the goal of tracking concentrations over time. Other sources of nondifferential error by time, including random error or individual variation attributable to toxicokinetic factors independent from age, sex, race/ethnicity, renal function, or iron status could potentially bias the trend estimates toward the null. Consequently, having individual repeated measurements could help to improve the estimates. Additionally, the variables used to measure smoking and exposure to tobacco smoke may not fully capture lifetime cumulative exposure to tobacco; therefore, the proportion of the decline in cadmium concentrations attributed to smoking may be biased.

Finally, our analysis has important strengths, including the large sample size that enabled us to conduct interaction models by survey and cadmium-determinant subgroups, including race/ethnicity; the availability of detailed information on relevant cadmium determinants, such as smoking; the standardization of the study protocol; the extensive laboratory quality control; and the representativeness of the study sample.

## Conclusion

Cadmium exposure, as measured in urine, substantially decreased in U.S. adults from 1988 to 2008. Our data suggest that declining smoking rates and exposure to tobacco smoke have contributed to reducing cadmium exposure in the United States, and that fewer people have urine cadmium at levels above the reference point proposed by the European Food Safety Authority (EFSA 2011) for the estimation of the tolerable weekly intake. However, cadmium remains a concern because of evidence indicating toxicity at the current levels of environmental exposure. For example, even at the reduced recent levels of exposure, cadmium has been related to cardiovascular, bone, and kidney disease in studies of NHANES 1999–2008 data (Egwuogu et al. 2009; Gallagher et al. 2008; Navas-Acien et al. 2004, 2005, 2009; Peters et al. 2010; Tellez-Plaza et al. 2008, 2010), supporting the need to further reduce cadmium exposure. Because the epidemiologic evidence available is mostly cross-sectional, prospective epidemiologic studies are needed to understand the health consequences of cadmium exposure and to evaluate the adequacy of current food and environmental safety standards. Additional public health efforts, including tobacco control interventions and efforts to reduce levels of cadmium in air, soils, and food, are critical to further prevent cadmium exposure in the general population.

## Supplemental Material

(246 KB) PDF
