# Supplementary material for: Reduction in Cadmium Exposure in the United States Population, 1988–2008: The Contribution of Declining Smoking Rates
Source: Environ Health Perspect. 2011 Nov 7;120(2):204–9. doi: 10.1289/ehp.1104020 (PMC3279452; doi:10.1289/ehp.1104020)
Supplement: (246 KB) PDF [file ehp.1104020.s001.pdf]

SUPPLEMENTAL MATERIAL

TITLE: "Reduction in Cadmium Exposure in the United States Population, 1988-2008: The Contribution of Declining Smoking Rates"

AUTHORS: Maria Tellez-Plaza, Ana Navas-Acien, Kathleen L. Caldwell, Andy Menke, Paul Muntner and Eliseo Guallar.

TABLE OF CONTENTS

Supplemental Material, Table 1.. page 2

Supplemental Material, Table 2.. page 3

Supplemental Material, Table 3.. page 5

Supplemental Material, Table 4.. page 6

Supplemental Material, Figure 1.. page 7

Supplemental Material, Table 1. Participant characteristics[a] by National Health and Nutrition Examination Survey phase groups

Characteristics	1988-94 N=13,691	1999-2002 N= 2,131	2003-2008 N= 3,937
Age (years)	44.2 (0.4)	46.3 (0.6)	47.5 (0.4)
Gender (% male)	47.9 (0.5)	47.1 (1.7)	46.4 (0.9)
Education < High School (%)	23.6 (1.0)	21.7 (1.1)	18.9 (1.1)
Race/ethnicity (%)			
White	76.6 (1.3)	72.6 (1.9)	72.9 (2.0)
African-American	10.2 (0.6)	9.9 (1.3)	10.7 (1.1)
Mexican-American	5.1 (0.4)	6.7 (0.8)	7.7 (1.0)
Other	8.1 (0.9)	10.8 (1.8)	8.7 (0.9)
BMI (Kg/m^2)	26.5 (0.1)	28.0 (0.2)	28.4 (0.2)
Smoking status (%)			
Never	46.0 (0.9)	54.0 (1.8)	53.8 (1.2)
Former	23.5 (0.6)	22.3 (1.3)	22.3 (0.9)
Current	30.5 (0.9)	23.7 (1.7)	24.0 (1.1)
Cigarette pack-year	12.9 (0.4)	9.3 (0.5)	10.1 (0.4)
Serum cotinine (ng/mL)	1.44 (1.23, 1.70)	0.40 (0.29, 0.55)	0.34 (0.27 0.43)
Ever cadmium-associated occupation[b] (%)	13.6 (0.5)	10.9 (0.8)	16.6 (1.2)
Duration of longest cadmium-associated occupation[c] (years)	13.5 (0.5)	14.6 (1.0)	15.1 (1.5)
Urine cadmium (µg/L)	0.36 (0.34, 0.38)	0.28 (0.27, 0.30)	0.25 (0.23, 0.26)
Urine cadmium (µg/g creatinine)	0.36 (0.34, 0.38)	0.28 (0.26, 0.29)	0.26 (0.25, 0.27)

Abbreviations: BMI, body mass index

[a]Percentages (standard errors) for categorical variables or means (standard errors) for continuous variables, except for serum cotinine, blood lead and cadmium, urine cadmium and creatinine-adjusted cadmium for which geometric means (confidence intervals) are reported. All the estimates were obtained by incorporating the complex survey design and weights in the analysis and are representative of the US population.

[b]Information available only until 2004 (1988-94 N = 13,534; 1999-2002 N = 2,126; 2003-2004 N = 1,206).

[c]Among individuals with a cadmium-associated occupation (1988-94 N = 1,827; 1999-2002 N = 251; 2003-2004 N = 207).

Supplemental Material, Table 2. Ratios (95% confidence intervals) of creatinine-corrected urine cadmium geometric means[a,b] by participant characteristics within time period

Characteristics	N	1988-94 GM Ratio (95% CI)	N	1999-2002 GM Ratio (95% CI)	N	2003-2008 GM Ratio (95% CI)
Age group, years						
< 35	4,305	1.00 (Referent)	478	1.00 (Referent)	893	1.00 (Referent)
35-50	3,613	1.83 (1.72, 1.94)	612	1.78 (1.63, 1.95)	1,006	1.78 (1.67, 1.91)
50-65	2,618	3.02 (2.77, 3.28)	489	2.67 (2.34, 3.05)	938	2.66 (2.40, 2.96)
≥ 65	3,155	3.46 (3.20, 3.74)	552	2.90 (2.69, 3.13)	1,100	3.21 (2.97, 3.47)
Sex						
Men	6,407	1.00 (Referent)	1,044	1.00 (Referent)	1,909	1.00 (Referent)
Women	7,284	1.39 (1.33, 1.46)	1,087	1.44 (1.35, 1.53)	2,028	1.36 (1.29, 1.43)
Race/ethnicity						
White	5,692	1.00 (Referent)	1,046	1.00 (Referent)	1,996	1.00 (Referent)
African-American	3,635	1.15 (1.07, 1.25)	382	1.03 (0.90, 1.18)	813	1.00 (0.93, 1.07)
Mex-American	3,792	1.06 (0.97, 1.15)	536	1.12 (1.00, 1.25)	733	1.06 (0.98, 1.15)
Other	572	1.22 (1.09, 1.37)	167	1.26 (1.09, 1.45)	395	1.20 (1.07, 1.35)
Education						
≥ High School	8,311	1.00 (Referent)	1,407	1.00 (Referent)	2,767	1.00 (Referent)
< High School	5,380	1.32 (1.25, 1.40)	724	1.28 (1.18, 1.40)	1,170	1.22 (1.16, 1.30)
BMI, kg/m^2						
<25	5,366	1.00 (Referent)	657	1.00 (Referent)	1,194	1.00 (Referent)
25 - 30	4,796	0.98 (0.93, 1.03)	801	0.83 (0.76, 0.91)	1,399	0.90 (0.83, 0.96)
≥ 30	3,529	0.91 (0.85, 0.97)	673	0.78 (0.69, 0.89)	1,344	0.80 (0.75, 0.86)
Smoking						
Never	6,782	1.00 (Referent)	1,174	1.00 (Referent)	2,151	1.00 (Referent)
Former	3,101	1.43 (1.33, 1.53)	494	1.29 (1.17, 1.41)	929	1.28 (1.21, 1.37)
Current	3,808	2.23 (2.10, 2.36)	463	2.23 (2.01, 2.47)	857	2.11 (1.96, 2.27)
Cigarette pack-years						
0	6,815	1.00 (Referent)	1,189	1.00 (Referent)	2,173	1.00 (Referent)
0 - 10	2,945	1.30 (1.20, 1.40)	398	1.37 (1.23, 1.53)	732	1.22 (1.14, 1.31)
10 - 20	1,277	1.93 (1.76, 2.11)	185	1.80 (1.58, 2.04)	341	1.78 (1.63, 1.94)
> 20	2,654	2.70 (2.56, 2.86)	359	2.40 (2.18, 2.65)	691	2.54 (2.37, 2.72)
Serum cotinine, ng/mL						
<0.05	1,728	1.00 (Referent)	955	1.00 (Referent)	1,895	1.00 (Referent)
0.05-10	8,395	1.26 (1.14, 1.39)	722	1.08 (0.98, 1.20)	1,211	1.09 (1.03, 1.16)
10-200	1,517	1.80 (1.61, 2.02)	220	1.84 (1.65, 2.06)	325	1.71 (1.53, 1.92)
≥200	2,051	3.02 (2.72, 3.35)	234	2.64 (2.36, 2.96)	506	2.35 (2.18, 2.52)
Duration of longest cadmium-associated occupation, years[c]						
0	11,921	1.00 (Referent)	1,778	1.00 (Referent)	969	1.00 (Referent)
0 - 10	584	1.21 (1.10, 1.32)	71	1.20 (0.98, 1.48)	64	1.31 (1.12, 1.54)
10 - 20	455	1.33 (1.19, 1.48)	64	1.34 (1.07, 1.67)	41	1.40 (1.10, 1.78)
> 20	513	1.43 (1.27, 1.61)	86	1.30 (1.06, 1.60)	83	1.23 (1.02, 1.50)

Abbreviations: BMI, body mass index; GM, geometric mean

[a]Models were adjusted for age (restricted cubic splines with 5 knots), sex (male, female), and race/ethnicity (white, black, Mexican-American, other).

[b]We obtained adjusted ratios of geometric means comparing cadmium determinants categories with respect to the reference category (i.e. in age subgroups the comparison is 35-50, 50-65 and ≥ 65 categories versus < 35 years) by exponentiating linear combinations of beta coefficients from regression models with log-transformed cadmium as the dependent variable and survey phase group and confounder factors as interacting independent variables. The ratio of the geometric means is interpreted as the factor by which a given category has a higher (ratio >1) or lower (ratio < 1) geometric mean compared to the reference category. For instance, in NHANES 1988-94, urine cadmium geometric mean in participants 35-50 years of age was 1.83 times (or 83%) higher compared to the geometric mean in participants <35 years of age, after adjustment for sex and race-ethnicity.

[c] Occupation variables were only available through 2004.

Supplemental Material, Table 3. Geometric means of urine cadmium levels by smoking status, age, gender and time period

	Never smokers					
	Male			Female		
Age group, years	1988-94	1999-02	2003-08	1988-94	1999-02	2003-08
<35	0.12	0.11	0.10	0.21	0.16	0.16
35-50	0.19	0.14	0.14	0.32	0.28	0.24
50-65	0.23	0.23	0.19	0.50	0.40	0.35
≥ 65	0.34	0.26	0.25	0.66	0.42	0.40
	Former smokers					
	Male			Female		
Age group, years	1988-94	1999-02	2003-08	1988-94	1999-02	2003-08
<35	0.17	0.16	0.12	0.25	0.20	0.14
35-50	0.30	0.21	0.18	0.40	0.34	0.27
50-65	0.49	0.33	0.30	0.83	0.44	0.46
≥ 65	0.61	0.44	0.43	0.94	0.67	0.61
	Current smokers					
	Male			Female		
Age group, years	1988-94	1999-02	2003-08	1988-94	1999-02	2003-08
<35	0.23	0.16	0.14	0.32	0.29	0.19
35-50	0.56	0.41	0.39	0.64	0.52	0.48
50-65	0.95	0.72	0.58	1.33	0.94	0.70
≥ 65	1.11	0.89	0.74	1.44	1.29	1.16

Supplemental Material, Table 4. Prevalence of participants with urine cadmium concentrations > 1µg/g by participant characteristics within time period [a]

	Prevalence of urine cadmium > 1µg/g, %		
Characteristics	1988-94	1999-02	2003-08
Overall	16.04	4.53	4.56
Age group			
<35	3.13	0.11	0.06
35-50	11.86	3.36	3.88
50-65	29.87	10.82	8.41
≥65	32.13	10.45	13.26
Sex			
Men	10.91	3.24	2.86
Women	20.64	5.81	6.21
Race/ethnicity			
White	15.43	4.67	4.57
African-American	15.69	3.53	2.49
Mexican-American	14.56	4.67	2.75
Other	23.32	4.61	8.44
Education			
≥ High School	14.28	3.72	4.09
< High School	21.48	7.82	7.09
BMI			
<25 kg/m^2	19.57	7.48	7.18
25 - 30	15.16	3.38	3.56
≥ 30	10.37	2.69	2.81
Smoking			
Never	4.84	0.95	0.88
Former	11.44	2.06	2.73
Current	36.52	16.28	13.84
Pack-years			
0	6.35	1.29	1.19
0 - 10	10.47	1.53	3.09
10 - 20	21.24	6.48	6.87
> 20	39.53	16.31	15.42
Serum cotinine, ng/mL			
<0.05	5.01	1.06	1.30
0.05-10	7.76	1.83	1.84
10-200	22.45	11.06	10.48
≥200	45.97	22.44	16.21
Duration of longest cadmium-associated occupation, years [b]			
0	15.00	3.80	4.24
0 - 10	20.29	8.47	4.81
10 - 20	23.52	14.42	9.18
> 20	25.07	10.54	4.13

Abbreviations: BMI, body mass index. [a] Prevalences of participants with urine cadmium concentrations > 1µg/g were adjusted for age (years modeled as restricted cubic splines with 5 knots), sex (men, women), and race/ethnicity (White, African-American, Mexican-American and other) and recalibrated to overall prevalence. [b] Occupation variables were only available through 2004

Supplemental Material, Figure 1. Age-adjusted geometric mean urine cadmium levels by sex and smoking status in NHANES 1988-2008.

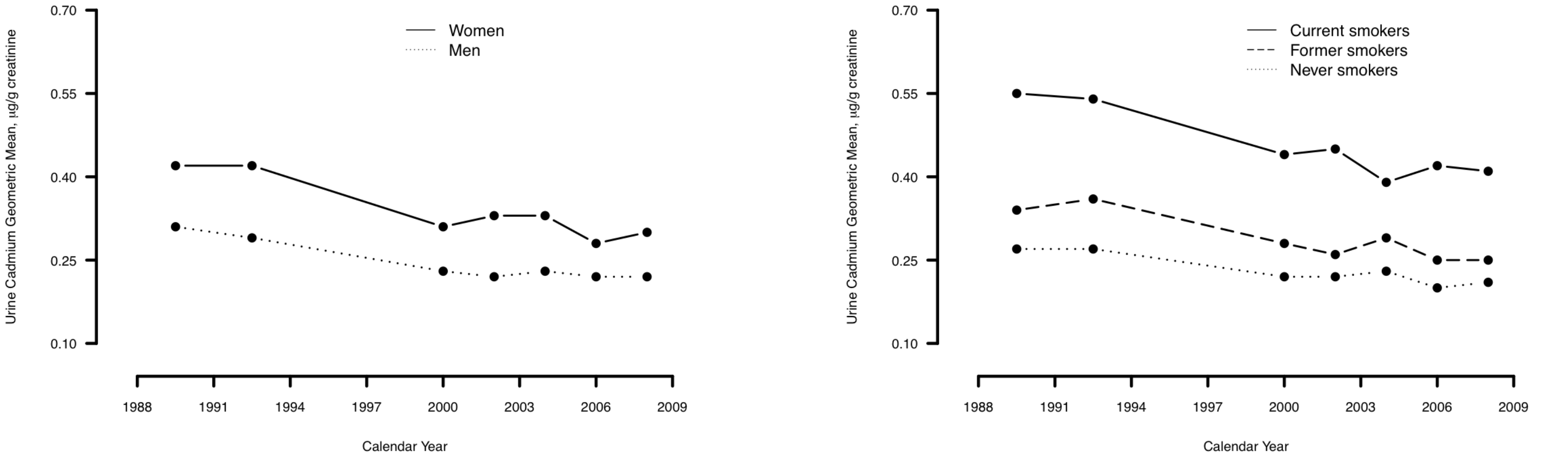

Abbreviations: NHANES, National Health and Nutrition Examination Survey.
Geometric means were adjusted for age (years modeled as restricted cubic splines with 5 knots) in survey wave, sex and smoking status specific strata.
